# Comparing the Therapeutic Potential of Stem Cells and their Secretory Products in Regenerative Medicine

**DOI:** 10.1155/2021/2616807

**Published:** 2021-08-19

**Authors:** Jhi Biau Foo, Qi Hao Looi, Pan Pan Chong, Nur Hidayah Hassan, Genieve Ee Chia Yeo, Chiew Yong Ng, Benson Koh, Chee Wun How, Sau Har Lee, Jia Xian Law

**Affiliations:** ^1^School of Pharmacy, Faculty of Health and Medical Sciences, Taylor's University, 47500 Subang Jaya, Selangor, Malaysia; ^2^Centre for Drug Discovery and Molecular Pharmacology (CDDMP), Faculty of Health and Medical Sciences, Taylor's University, 47500 Subang Jaya, Selangor, Malaysia; ^3^My Cytohealth Sdn Bhd, Bandar Seri Petaling, 57000 Kuala Lumpur, Malaysia; ^4^National Orthopaedic Centre of Excellence for Research and Learning (NOCERAL), Department of Orthopaedic Surgery, Faculty of Medicine, Universiti Malaya, 50603 Kuala Lumpur, Malaysia; ^5^Institute of Medical Science Technology, Universiti Kuala Lumpur, 43000 Kajang, Selangor, Malaysia; ^6^Centre for Tissue Engineering and Regenerative Medicine, Faculty of Medicine, Universiti Kebangsaan Malaysia Medical Centre, Jalan Yaacob Latif, 56000 Kuala Lumpur, Malaysia; ^7^School of Pharmacy, Monash University Malaysia, 47500 Bandar Sunway, Selangor, Malaysia; ^8^School of Biosciences, Faculty of Health and Medical Sciences, Taylor's University, 47500 Subang Jaya, Malaysia

## Abstract

Cell therapy involves the transplantation of human cells to replace or repair the damaged tissues and modulate the mechanisms underlying disease initiation and progression in the body. Nowadays, many different types of cell-based therapy are developed and used to treat a variety of diseases. In the past decade, cell-free therapy has emerged as a novel approach in regenerative medicine after the discovery that the transplanted cells exerted their therapeutic effect mainly through the secretion of paracrine factors. More and more evidence showed that stem cell-derived secretome, i.e., growth factors, cytokines, and extracellular vesicles, can repair the injured tissues as effectively as the cells. This finding has spurred a new idea to employ secretome in regenerative medicine. Despite that, will cell-free therapy slowly replace cell therapy in the future? Or are these two modes of treatment still needed to address different diseases and conditions? This review provides an indepth discussion about the values of stem cells and secretome in regenerative medicine. In addition, the safety, efficacy, advantages, and disadvantages of using these two modes of treatment in regenerative medicine are also critically reviewed.

## 1. Introduction

Cellular therapy, also known as “cell-based therapy,” involves the transplantation of human cells to stimulate the regeneration of damaged tissues and modulate the mechanisms underlying disease initiation and progression. Multiple types of human cells, including stem cells and progenitor cells, have been used to treat different diseases. Stem cell therapies using induced pluripotent stem cells (iPSCs) [1]_,_ embryonic stem cells (ESCs) [2], and adult stem cells such as mesenchymal stem cells (MSCs) [3] have been tested preclinically and clinically for years. Nowadays, MSC is widely used in the field of tissue engineering and regenerative medicine. In general, stem cell therapy has grown to become an attractive option to reduce the overall need for tissue transplantation and minimize the waiting time for patients [4]. Numerous clinical studies have indicated that stem cell administration is a safe and promising therapeutic approach. The transplanted cells can differentiate to restore the structure and function of injured tissues [5, 6]. However, more and more evidence suggested that the transplanted cells promote tissue regeneration mainly through paracrine secretion.

Recent studies have shown that the transplanted cells secrete paracrine factors that directed the proliferation and differentiation of surrounding cells as well as produce chemoattractants that attracted the migration of effector cells to the injured sites. The “cell-free therapy” that utilizes the therapeutic molecules, i.e., secretome, secreted by stem cells has become more popular as it offers many advantages and avoids many limitations bothering the cell-based therapy. The composition of secretome is very dynamic, depending on the cell type and stimulus from their surrounding microenvironment [7]. Generally, stem cell-secreted secretome comprises (i) a complex mixture of soluble components such as growth factor and cytokines (obtained as the conditioned medium), (ii) a vesicular portion composed of extracellular vehicles (EVs), and (iii) cell organelles (e.g., mitochondria). It has been suggested that secretome can promote cell-cell communication, interact with other cells in their immediate environment, and transfer functional biomolecules to initiate tissue repair or regeneration. Generally, secretome has been found to possess proangiogenic, antiapoptotic, antifibrotic, anti-inflammatory, immunomodulatory, and proproliferative properties [8–10]. Nevertheless, extensive investigations are still required to better understand the therapeutic mechanism of secretome transplantation, its safety issues, and the clinical efficacy, mainly through clinical trials. In this review, the focus is on the values of stem cells and secretome in regenerative medicine, as well as discussing the latest insights on the safety, efficacy, advantages, and disadvantages of using these two modes of treatment.

## 2. Classification of Cell-Based Therapy

### 2.1. Stem Cell Therapy

Stem cell therapy can be categorized into autologous and allogeneic based on the tissue donor. To date, autologous stem cell transplantation has been performed for a broad range of purposes, such as to promote cardiac and cartilage regeneration, expedite wound healing, and improve aesthetic appearance. Autologous stem cells are used as they are readily available from many tissue sources and have a lower risk of life-threatening complications such as graft-versus-host disease (GVHD), free of ethical issues, and nonimmunogenic. The adverse events reported in the transplantation of stem cells are most likely unrelated to the treatment but to the underlying disease instead [11].

Allogeneic stem cell transplantation is gaining more attention in the past decade due to its advantages, such as reduction of functional variability through the pooling of cell products from multiple donors in a master bank, and it is readily available off-the-shelf for clinical applications. Allogeneic bone marrow-derived MSCs (BMSCs) mixed with autologous chondrocytes have been transplanted into the knee joint of patients with symptomatic cartilage defect, and the results showed the regeneration of hyaline cartilage with a high concentration of proteoglycans and type II collagen at 12 months [12]. A clinical trial on end-stage liver cirrhosis also revealed that allogeneic stem cell transplantation positively affects the patients' condition by improving the serum albumin levels and model for end-stage liver disease (MELD) scores after six months [13]. Paul et al. reported the immunomodulatory benefit of allogeneic MSC infusion by reducing the rejection of transplanted corneal during the immediate posttransplant period [14].

### 2.2. Stem Cell Derivatives/Secretome

**Figure 1:**
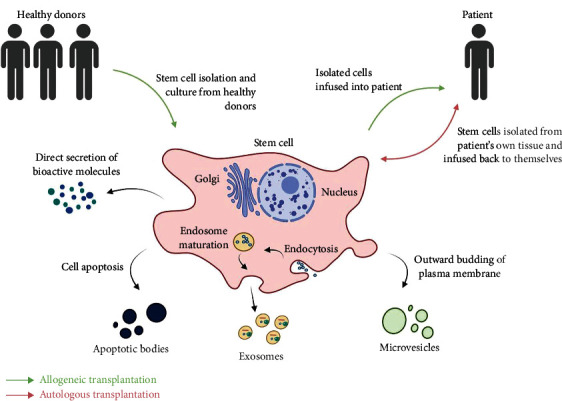
Classification of stem cell therapy and stem cell-derived secretome. Autologous stem cell transplantation involves the isolation of stem cells from the patient and infusion back to the same patient during treatment. Whereas in allogeneic stem cell transplantation, stem cells from single or multiple healthy donors are given to the patient. Stem cell secretome consists of bioactive molecules (including growth factors, cytokines, chemokines, enzymes, extracellular matrix) secreted directly out to the cell microenvironment or encapsulated within the extracellular vesicles that can be classified into three groups: apoptotic bodies which form during cell apoptosis, exosomes as the product of endosome maturation, and microvesicles by outward budding of the plasma membrane (created with BioRender.com).

In the field of regenerative medicine, the therapeutic effects of stem cells are not constricted to cell-cell interactions. A broad range of bioactive molecules is found in stem cell secretion, including growth factors, cytokines, chemokines, enzymes, extracellular matrix (ECM), and extracellular vesicles (EVs), collectively known as the secretome [15]. The secretome is a crucial component in exhibiting the therapeutic effect of stem cells (Figure 1).

## 3. Stem Cells

### 3.1. Source of Stem Cells

The collection of pluripotent stem cells (PSCs) such as ESCs is ethically controversial as it involves the destruction of possible human life. Furthermore, ESC is also considered an allogeneic source of cells which may cause immune incompatibility. However, there is an immediate solution to avoid ethical repercussions; the adult somatic cells can be reprogrammed into iPSCs which essentially functionally behaved as ESCs [16]. Genetically modified PSC is utilized in disease modeling to overcome the species-specific differences as observed in an animal model. It also serves as a potential cure to a currently permanent condition such as thyroid disease [17–19], cardiovascular disease [20], macular degeneration [21, 22], or Parkinson's disease that only can be managed with lifelong medications. Unfortunately, PSC has raised safety concerns as some research reported tumorigenicity [23] or epigenetic aberrations posttreatment [24–26]. A fail-safe suicide gene known as inducible caspase-9 (iCasp9) has been tested extensively in vitro and in vivo as a potentially viable solution to remove the residual pluripotent cell that may cause teratoma formation [27, 28].

On the other hand, multipotent cells have a narrower spectrum of differentiation than pluripotent stem cells and can differentiate into discrete cells of specific lineages. Examples of multipotent cells are hematopoietic stem cells (HSCs) and MSCs. HSCs can be isolated from peripheral blood, bone marrow, and umbilical cord blood, whereas MSCs can be found in bone marrow, umbilical cord, cord blood, placental, peripheral blood, adipose tissue, dental tissues, skin, salivary gland, and synovial fluid [29–32]. Although there are variations in molecular composition, surface antigen expression, differentiation capacity, and immunomodulatory property in MSCs isolated from different tissue sources [23, 33], however, functional analyses showed that all the secretome have similar functionality, i.e., to promote cell migration and inhibit cell apoptosis [34].

### 3.2. Mechanism of Therapeutics

**Figure 2:**
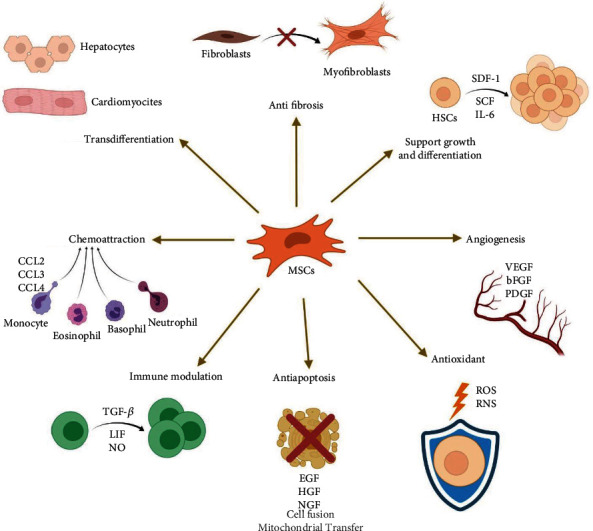
Mechanism of action of MSCs in tissue repair and immunomodulation. MSCs exert its therapeutic effects via various modulators. SDF-1: stromal cell-derived factor 1; SCF: stem cell factor; IL-6: interleukin-6; VEGF: vascular endothelial growth factor; bFGF: basic fibroblast growth factor; PDGF: platelet-derived growth factor; ROS: reactive oxygen species; RNS: reactive nitrogen species; EGF: epidermal growth factor; HGF: hepatocyte growth factor; NGF: nerve growth factor; TGF-*β*: transforming growth factor-beta; LIF: leukemia inhibitory factor; NO: nitric oxide; CCL: C-C motif chemokine ligand (created with BioRender.com).

MSCs were thought to promote tissue regeneration via transdifferentiation to replace the damaged cells and cell fusion to save the dying cells. However, many studies have found that these mechanisms are insufficient, and MSCs seem to secrete a myriad of paracrine factors, e.g., growth factors, chemokines, and cytokines, to promote tissue regeneration and modulate the immune response. This notion is supported by the low engraftment of the transplanted cells at the target site and rapid loss of the transplanted cells in vivo. The mechanisms employed by MSCs in tissue repair and immunomodulation have been excellently reviewed in previous publications [35–39]. In this section, we will only provide a glimpse at their mechanism of action in a short summary. In brief, MSCs secrete anti-inflammation, antiapoptosis, antioxidative, antifibrosis, proangiogenesis, promitosis, and chemotactic factors to stimulate tissue regeneration (Figure 2).

### 3.3. Multifactorial Crosstalk

#### 3.3.1. Direct Signaling

Cell-cell signaling by direct contact allows stem cells to communicate and respond to other cells. It is not always necessary as stem cells have other soluble-dependent crosstalk as well. In a mixed immune cell culture such as peripheral blood mononuclear cells (PBMCs), cell contact is not required for MSCs to exert their anti-inflammatory effect. Contrastingly, when MSCs were introduced to a lymphocyte-only culture and cell-cell contact was prohibited, MSCs failed to induce FoxP3 and CD25 expressions in CD4^+^ T cells [40]. The adhesion molecules ICAM-1 and VCAM-1 will not form in the lack of direct MSC-lymphocyte contact [41]. Moreover, MSCs require direct contact with immune cells to upregulate cell-surface proteins such as programmed death-ligand 1 (PD-L1) and Fas ligand to suppress inflammation [42, 43]. As a result, the immunoregulatory properties of MSC will not be exerted to their full potential. The modulation of dendritic cell maturation by MSCs also requires both direct cellular contact and the soluble factor, interleukin (IL)-6. Loibl et al. reported better results when endothelial progenitor cells were cocultured with MSCs as it significantly upregulated the mature endothelial cell marker, PECAM-1, relative to the transwell setup [44]. The immunosuppression of B cells was more efficient in direct cocultured with MSCs [45].

#### 3.3.2. Secondary Crosstalk

Paracrine signaling is the main mechanism of MSC therapy. It was initially thought that MSC would migrate and engraft at the site of injury. Nonetheless, most of the MSC administrated intravenously are sequestered in the vasculature of the lungs, with only a few MSCs homed to the tissue of interest. Studies have also noted that exogenous MSCs unable to retain their population long enough to completely replace the affected tissue. Hence, the lasting reparative effect of MSCs is largely attributed to its ability to secrete trophic factors to ameliorate the inflammation in other parts of the body [35, 46–48]. Chin et al. claimed that the anti-inflammatory cytokine levels remained elevated from baseline up until six months post-MSC transfusion [46]. MSCs are known to secrete immunosuppressive factors such as transforming growth factor-beta (TGF-*β*), vascular endothelial growth factor (VEGF), IL-10, prostaglandin E2 (PGE2), indoleamine 2,3-dioxygenase (IDO), and galectin-1 into the circulatory system [47–51]. These molecules interact with the immune cells such as T and B cells to suppress their proliferation and differentiation, causing the polarization of macrophage to an anti-inflammatory phenotype and reduction of the pro-inflammatory milieu consists of cytokines such as tumor necrosis factor-alpha (TNF-*α*), interferon-gamma (IFN-*γ*), and IL-6 [46, 47, 49, 52–55].

#### 3.3.3. Necrobiology

Necrobiology is a term used to describe the life processes associated with morphological, biochemical, and molecular changes related to cell death and the consequences and tissue response to cell death [56]. It encompasses four mechanisms by which derivatives of MSCs can retain significant clinical efficacy, including apoptosis, autophagy, mitochondrial transfer, and extracellular vesicle production [57]. The bioactive parts of dead or dying MSCs can trigger immunomodulatory properties in the host without the concern over cell survival and the formation of large aggregates [58–60].

*(1) Apoptosis*. Apoptosis of cultured MSCs can be induced via nutrient deprivation. In addition, some studies found that IFN-*γ* and TNF-*α* also can trigger MSC apoptosis through the nitric oxide (NO) [61] and Fas [62] pathways, respectively. One can consider inhibiting NO to prolong MSC survival, noting that it also will restrict the immunosuppression capacity of MSCs on the lymphocytes. Interestingly, Mancuso et al.'s study of knee osteoarthritis using an in vitro model revealed that apoptotic MSCs were more immunosuppressive than healthy MSCs [63]. Moreover, Chang et al. found that apoptotic MSCs were more effective in attenuating organ damage in rat sepsis models compared to the healthy MSCs [64]. Cheung et al. found that monocytes that efferocytosed the apoptotic MSCs have higher expression of IDO, PD-L1, and cyclooxygenase 2 (COX2), and these cells secrete more PGE2 and IL-10 as well as lower TNF-*α* to subside the inflammation. Then, they proceeded to monitor the serum PGE2 levels of eight patients with severe steroid-resistant GVHD who received MSC therapy. It was found that the responders demonstrated increment in PGE2 levels while the nonresponders showed not changes in PGE2 levels. Albeit, in a small sample size of eight GVHD patients, this study teased the possibility of apoptotic MSCs in translational medicine [65].

*(2) Autophagy*. In terms of stem cells, autophagy plays a pivotal role in maintaining genomic stability and retain its potency and differentiation capacity [66]. Gao et al. discovered that autophagy may regulate MSC immunoregulation through TGF-*β*1 signaling. The proliferation of CD4^+^ T helper cells was inhibited when cocultured with autophagic MSCs. However, when autophagy is inhibited, MSCs failed to suppress the proliferation of T cells [67]. Additionally, autophagy activation in MSC transplantation has protective effects on the damaged tissues. These protective effects of autophagy can be reversed using autophagy inhibitors such as 3-methyladenine and chloroquine. Autophagy can be induced by hypoxia and nutrient depletion, and it has been shown to protect MSCs in vitro [68]. Furthermore, Zhang et al. showed that hypoxic preconditioning on MSCs can enhance its functional survival to restore cardiac function in ischemia models [69]. Using rapamycin to induce the autophagy mechanism, Wang et al. showed that MSC-derived exosomes prevented acute kidney injury caused by cisplatin [70]. Similarly, Hou et al. induced autophagy by pretreating MSCs with starvation and rapamycin. In their study, it was shown that autophagy prevented the autophagic MSCs from irradiation injury and maintained the stemness after exposure to reactive oxygen species- (ROS-) induced damage [71]. Park et al. attributed the neuroprotective effects of MSCs to the higher levels of autophagy in a parkinsonian mice model and MPP^+^ treated neuronal cell culture [72].

*(3) Mitochondrial Transfer*. MSCs are known to reprogram the host cells by the transfer of mitochondria. It is a process that requires direct cell-cell contact through tunneling nanotubes (TNT) or gap junctions [73]. Mitochondria also can be transferred via secreted EVs [56]. The mitochondrial transfer has a prominent role in protecting the recipient cells from oxidative stress, radiation injury, and hypoxic injury as well as recovering the mitochondrial membrane potential and aerobic respiration and modulating the host immune response [74, 75]. Upregulation of Miro 1, a mitochondrial Rho-GTPase, has been reported to enhance mitochondrial transfer, subsequently improve the MSC therapeutic efficacy [76].

*(4) Extracellular Vesicles*. According to MISEV 2018, EVs are nonreplicating particles of size 100-200 nm and encapsulated by a lipid bilayer [77]. MSC-derived EVs contain bioactive molecules including genetic materials, microRNAs, enzymes, signaling proteins, immunomodulatory factors, and growth factors [78]. EVs have the potential to be developed into cell-free therapy with the benefits of MSC immunomodulation but without the concerns of maintaining the cell viability or risk of immune rejection in allogeneic transplantation. Many studies showed that MSC-derived EVs are as effective as MSCs in treating diseases [79–81]. Apoptotic cells are known to produce different types of EVs and apoptotic bodies that can influence the surrounding cells. Apoptotic cell-derived EVs are rich in spliceosomes that alter the RNA splicing in recipient cells [82]. More data are showing that apoptotic cell-derived EVs play a significant role in immune modulation in autoimmunity, infection, and cancer, implicating that they are not just cell debris [83]. All these findings indicating that apoptotic cell-derived EVs could be an important medium of communication between the dead and living cells [84]. Nonetheless, to date, apoptotic cell-derived EVs are not well studies. Thus, what we know is still very limited.

## 4. Secretome

**Figure 3:**
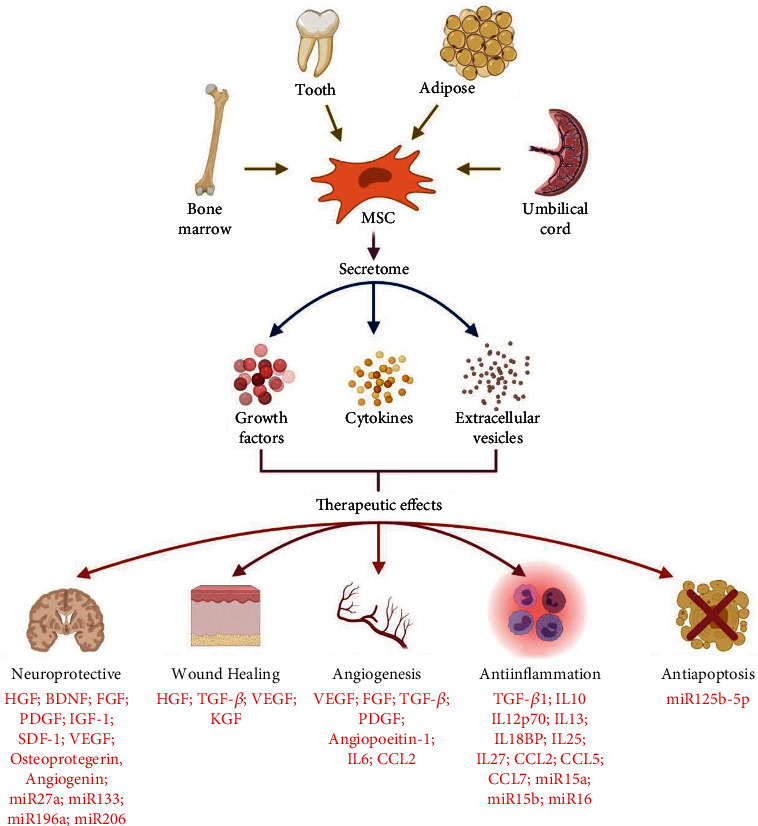
Mesenchymal stem cell sources, secretome content, and its therapeutic effects. MSCs can be derived from various sources, including bone marrow, teeth (deciduous vs. nondeciduous), adipose tissue, and umbilical cord. MSCs mainly rely on the secretome, which consists of various soluble factors (growth factors and cytokines) and extracellular vesicles to exert their therapeutic effects. Neuroprotection, acceleration of wound healing, induction of angiogenesis, suppressing of inflammation, and prevention of cell apoptosis are some of the reported therapeutic potentials of MSC secretome. HGF: hepatocyte growth factor; BDNF: brain-derived neurotrophic factor; FGF: fibroblast growth factor; PDGF: platelet-derived growth factor; IGF-1: insulin-like growth factor 1; SDF-1: stromal cell-derived factor 1; VEGF: vascular endothelial growth factor; TGF-*β*: transforming growth factor-beta; KGF: keratinocyte growth factor; IL: interleukin; miR: microRNA; CCL: C-C motif chemokine ligand (created with BioRender.com).

Secretome is often referred as a group of biologically active molecules or factors that are released by cells into their extracellular environment [85]. Although MSCs derived from various anatomical sites may exhibit similar morphological and immunophenotypic characteristics, numerous evidence showed that they secrete a distinct set of secretome that is normally associated with the host age and specific microenvironment that the cells were grown. The secretome may even fluctuate in response to various physiological changes and pathological circumstances. In general, MSC secretome is made up of a variety of growth factors, cytokines, and EVs that conferred its tissue repair and regenerative potential, mainly attributed to their capability to stimulate cell proliferation, formation of new blood vessels, and their immunomodulatory effects (Figure 3) [85, 86].

### 4.1. Growth Factor

Different investigations have shown that the growth factors present in MSC secretome may either work synergistically to exert their tissue regenerative potential or the presence of individual growth factors could be sufficient to achieve the desired therapeutic objective. For instance, brain injury such as stroke usually involves brain tissue damage due to a lack of blood supply. Hence, stroke therapy usually requires the promotion of new blood vessel formation and brain cell production, along with suppression of further cell death and inflammatory processes [85]. These have been successfully achieved via administration of BMSC and adipose tissue-derived MSC (AT-MSC) secretome that contain a mixture of hepatocyte growth factor (HGF), brain-derived neurotrophic factor (BDNF), fibroblast growth factors (FGF), and platelet-derived growth factor (PDGF) [7, 87]. Meanwhile, another study by Ding et al. has suggested a direct involvement of increased insulin-like growth factor-1 (IGF-1) levels that exhibited neuroprotective effect in a mouse model of brain stroke through regulating its ischemic and inflammatory condition to reduce the volume of brain infarct while improving the function of brain cells [88]. Similar observations also have been reported in Huntington's disease mouse model whereby transplantation of BMSCs led to elevated expression of stromal cell-derived factor-1 (SDF-1) to improve blood supply to the damaged brain striatum tissue via stimulation of angiogenesis [89]. The neuroprotective role of SDF-1 had also been verified in another rat model of Parkinson's disease whereby the grafted BMSCs inhibited apoptotic activities in the affected dopaminergic neuronal tissue, which significantly recovered the behavior of the diseased rats [90]. Besides brain injuries, therapeutic effects of growth factors present in MSCs have also been investigated for other pathologic conditions such as cutaneous injury, whereby the use of AT-MSC secretome that contains VEGF, HGF, transforming growth factor *β* (TGF-*β*), and keratinocyte growth factor (KGF) was able to induce greater cellular proliferation, trigger cell migration, and decrease the wound size at a faster rate [91–95]. The positive impact of these diverse growth factors in promoting angiogenesis [96], regenerating muscle tissue [97], and reducing incidences of premature infant diseases such as periventricular leukomalacia, retinopathy of prematurity, bronchopulmonary dysplasia, and necrotizing enterocolitis [98] also has been implicated.

### 4.2. Cytokines

Whilst growth factors are more frequently associated with induction of cellular proliferation or prevention of cell death for tissue regeneration, cytokines present in the MSC secretome play a more important role in regulating inflammatory activities in pathologic conditions to attain the therapeutic effect. In the MSC secretome, both anti-inflammatory cytokines (such as tumor necrosis factor *β*1 (TNF-*β*1), IL-10, IL-12 p70, IL-13, IL-18 binding protein, IL-25, and IL-27) and proinflammatory cytokines (such as TNF-*α*, interferon *γ* (IFN-*γ*), IL-1b, IL-6, IL-8, and IL-9) could be present. The impact of MSC secretome on the inflammatory process is usually governed by the balance of these anti- and proinflammatory cytokines [86]. For example, the destruction of pancreatic cells in autoimmune diabetes mellitus type 1 disease by proinflammatory cytokines, including TNF-*α*, IFN-*γ*, and IL-1b, could be reversed by treating the primary islet cells with MSC secretome containing significantly elevated levels of anti-inflammatory cytokines (IL-4 and IL-10), resulted in the prevention of cell apoptosis and improvement in insulin secretion [99]. The importance of anti-inflammatory cytokines produced by MSCs was further demonstrated by Hsu et al. who utilized MSCs to suppress inflammation-associated transplant arteriosclerosis through the secretion of IL-10 [100]. In another study by Ogata et al., MSC secretome was shown to stimulate bone healing in a rat bone defect model by increasing the migration of endogenous stem cells into the defect area. Subsequent analysis revealed the presence of various important cytokines in the MSC secretome which are essential to suppress inflammation as well as induce cell proliferation, angiogenesis, recruitment, and osteogenesis. These cytokines include chemokine ligand 2, chemokine ligand 5, chemokine ligand 7, and TNF-*β* [101].

### 4.3. Extracellular Vesicles

Other than growth factors and cytokines, EVs are another important subset of MSC secretome that play a crucial role in both normal and pathological processes through maintenance of homeostasis as well as regulation of immune function, tissue regeneration processes, and tumorigenesis. These EVs that carry therapeutic cargo, including nucleic acids, proteins, and lipids, are originally a method of communication between neighboring and distant cells. They can be divided into two types depending on their sizes, that is either exosomes (40-200 nm) or microvesicles (50-1000 nm) [4]. Example of the therapeutic proteins abundantly present in EVs secreted by MSCs includes osteoprotegerin and angiogenin that were found to be the key players for bone regeneration in a rat model of bone defect [101]. Besides proteins, microRNAs (miRNAs) inside the EVs secreted by BMSCs, such as miR27a, miR196a, and miR206, were also found to be crucial in triggering the expression of osteogenic genes for acceleration of bone regeneration in a rat model of calvarial bone defect [102]. On the other hand, miR133 is an important miRNA produced by MSCs to stimulate neuronal tissue remodeling in a rat model of stroke disease [103, 104]. Meanwhile, miR125b-5p is an example of miRNA that could exert antiapoptotic effect as demonstrated by its ability to suppress expression of proapoptotic BAK1 and p53 genes in a myocardial infarction model, hence preventing the death of cardiomyocytes and subsequently allowing the repair of the ischemic tissue [105]. Apart from modulation of tissue regeneration, some miRNAs are also able to regulate the immune system to suppress the extent of tissue injury. For instance, miR15a, miR15b, and miR16 could inhibit the expression of CX3CL1 to prevent recruitment of macrophages to the ischemic kidney, therefore reducing the inflammatory process in the injured kidney [106].

## 5. Delivery and Homing of Stem Cells and Secretome

Stem cells and secretome can be delivered via various routes of administration to elicit their therapeutic actions. Thus far, direct injection to the target site and intravenous injection is most widely used as they deliver the biologics to the target tissue more effective compared to other routes of administration.

Compared to exosomes, homing of stem cells to the target tissue is critical for the cells to exert their therapeutic effects. The efficacy of stem cell homing to the target tissue upon transplantation is very much dependent on the route of administration. There is an intrinsic relationship between different chemical factors and MSCs that influence its homing and reparative effects. Stromal-derived factor-1 (SDF-1)/CXC chemokine receptor 4 (CXCR4) axis is imperative in the recruitment of MSCs to the injured tissue [107, 108] and inadvertently promotes neovascularization [109, 110]. The increased expression of SDF-1 after tissue injury stimulates the expression of CXCR4 on MSCs which improve stem cell homing and engraftment to the injured site [111]. Besides, Qin et al. described that SDF-1 regulates the MSC immunomodulatory effects through CXC chemokine receptor 7 (CXCR7). In low concentration, the proliferation of MSCs is induced, and the regulatory B cells produce various cytokines including (IL-6, IL-10, IL-4, IFN-*γ*, TNF-*α*) [112]. Zheng et al. suspected enhanced homing of CXCR4-overexpressing MSCs to the site of colitis resulted in the significant reduction of tumor formation when compared to the untreated group [113]. Similarly, Wang et al. observed improvement in cell migration using CXCR4-overexpressing MSCs, and the progression of diabetic retinopathy was hampered [47]. Without CXCR4 gene transfection, MSC is considerably less effective in repairing cardiovascular damage as the necessary vascular cell adhesion molecule-1 (VCAM-1), and intercellular adhesion molecule-1 (ICAM-1) cannot be stimulated solely with SDF-1 [110, 114].

### 5.1. Direct Injection to the Target Tissue

Direct injection is a straightforward approach to deliver stem cells to the target tissue. For example, stem cells can be injected through the intraspinal and intrathecal route to treat spinal cord injury and intraarticularly to treat osteoarthritis [115, 116]. Direct injection can increase the homing of stem cells to the target tissue, and this is crucial as stem cells can self-renew and differentiate into the desired cells to repopulate and regenerate the injured or lost tissue. In addition, local delivery of stem cells to the target tissue is necessary as the secreted bioactive factors act in a paracrine manner and may be degraded in the bloodstream before reaching the target tissue when administered distantly. In the context of secretome such as exosomes, direct injection is applicable as well. MSC exosomes were effective in repairing critical size osteochondral defects in immunocompetent rats, as evidenced by the increased cellular proliferation and infiltration, enhanced matrix synthesis, and presence of regenerative immune phenotype [117]. These results are achieved via intraarticular injection of exosomes.

Even though many researchers have analyzed the therapeutic efficacy of different routes of delivery for stem cell therapy, however, there is still no congruous consensus on the optimal delivery method among the different reports [118]. Although direct injection of cell-based treatment (either stem cell or secretome) to the affected tissue is appealing and has long been documented, this approach may accompany problematic complications if it is not carefully planned, performed, or managed. The migration of stem cell or lymphatic drainage is the physiological process that would reduce the number of injected cells or quantity of secretome initially present in the target tissue vicinity. In addition, the hostile wound environment with intense inflammation is not ideal to support the survival of the transplanted cells [119].

### 5.2. Intravenous Administration

Delivery of cells through vein has been suggested in numerous preclinical studies and clinical trials [120]. Intravenous administration is advantageous because of its systemic distribution and ability to reach deeper tissues. However, intravenous administration also carries the risk of cell entrapment in the lung vasculature, and the retention time for the cells and their effects are short. The main concern of intravenous administration is to get enough cells to the target tissues. Harting et al. managed to infuse rats with MSCs intravenously to treat traumatic brain injury [121]. The group did not find cell homing to the target tissue. However, the rats still showed improvement in motor and cognitive functions. In terms of secretome, delivery via the intravenous route is safe due to the lower risk of embolism compared to the delivery of stem cells. In the case of neurological disorders, emboli of administered cells in the cerebral microvascular can exacerbate the disease and can be life-threatening. Intravenous administration of whole secretome or its components, i.e., exosomes, has been reported to be safe and capable to ameliorate several diseases (Table 1).

### 5.3. Scaffold

On top of that, new delivery strategies utilizing the biomaterials such as polymeric scaffolds and cell sheets can increase cell retention on top of providing a supporting matrix to enhance cell survival and functionality [131]. The polymeric scaffold stabilizes the stem cells and their soluble factors as well as permits sustained delivery of these bioactive factors. The structure also supports cell growth. The architecture of the scaffolds including stiffness and pore arrangement is an important regulator of stem cell differentiation. The microarchitecture of the scaffold has an impact on the differentiation of MSCs into cells of interest. Phadke et al. found the randomly oriented pores were better suited for osteogenic differentiation of MSCs when compared to the lamellar column-arranged pore network [132]. Multiple studies have focused on the development of insulin-producing cells to treat diabetes. Enderami et al. noted a significantly higher expression of glucose-regulating genes including Pdx1, insulin, glucagon, and Ngn3 genes in poly-L-lactic acid and polyvinyl alcohol (PLLA/PVA) 3D scaffold than in the regular 2D culture [133]. The 3D scaffold provides a supporting structure to maintain the cell-cell and cell-matrix interactions. The stem cells cultured in nanofibrous scaffolds generate pancreatic organoids which are morphologically and functionally similar to the mature pancreatic *β*-cells [134–136].

The main advantage of using cell sheets is the fabrication techniques that will not disrupt the cell-cell and cell-matrix contact [137]. Usage of cell sheets fabrication techniques such as temperature-responsive culture surface, photoresponsive polymer, and ultrasound irradiation enables the detached cells to maintain their cell surface proteins, cellular junctions, and extracellular matrix [138]. Cell sheets may be developed into an advanced cell delivery method for the treatment of many tissue injuries, including cardiovascular diseases, cutaneous wound healing, and tendon/ligament injuries. The combination of multiple cell sources in the fabrication of cell sheets may mimic the natural state of tissue to allow better grafting of cells and better tissue regeneration.

## 6. Stem Cells and Secretome Clinical Trials

### 6.1. Stem Cell Clinical Trials

Thus far, many clinical trials using MSCs have been completed, and some of the therapies, e.g., Cupistem®, Queencell®, Cartistem®, Cellgram®, Neurorata-R®, Prochymal®, Stempeucel®, and MesestroCell, have received market authorization in Korea, Canada, India, and Iran [139]. A list of worldwide clinical studies using stem cells in different phases can be found in National Institutes of Health Clinical Trials.gov website (https://www.clinicaltrials.gov/) (Table 2).

#### 6.1.1. Pluripotent Stem Cells

There are eight completed clinical trials on ESC transplantation, and seven of them are associated with eye disease and one for ischemic heart disease. The PSC clinical trials mainly focus on eye disease as the tissue is easily accessible for transplantation, and serious adverse events (SAEs) on the eye are less likely to be life-threatening. Furthermore, PSC transplantation is associated with higher risks of tumor formation. Since the eyeball is a confined space that has few vasculature connections with the rest of the body, the tumor is less likely to metastasize. However, none of the studies listed is presented with results. All these studies are either phase I or phase I/II, indicating that the translational research of PSC therapy is still in the early phase.

#### 6.1.2. Multipotent Stem Cells

There are more completed MSC clinical trials compared to the PSC clinical trials as the cells are safer and have fewer ethical concerns. Many MSC clinical trials have published their results. Generally, MSC therapy is found to be safe and well-tolerated by the patients. In addition, some studies also reported the efficacy of MSC therapy to treat a battery of diseases. MSC therapy has received market authorization in several countries for the treatment of diseases such as Crohn's fistula, cartilage defects, osteoarthritis, major adverse cardiac events, amyotrophic lateral sclerosis, aGvHD, and critical limb ischemia [139]. Very recently, a parallel assigned controlled, nonrandomized phase I clinical trial has been conducted to evaluate the safety of human umbilical cord-derived mesenchymal stem cell (UC-MSC) infusion to treat patients with moderate and severe COVID-19 pulmonary disease [140]. Eighteen hospitalized COVID-19 patients were enrolled on the study, and nine of them received three cycles of intravenous infusion of UC-MSCs (3 × 10^7^ cells/infusion). Twenty-eight days after the first infusion, no UC-MSC infusion associated SAEs were observed except for one patient in the treatment group that required mechanical ventilation compared to four patients in the control group. All patients recovered following the treatment and were discharged. These data showed that intravenous UC-MSC infusion is safe and well-tolerated in patients with moderate and severe COVID-19.

### 6.2. Secretome Clinical Trials

Clinical trials of cell-free therapy are taking the emerging field from basic science to clinical application. Numerous trials are/have been conducted for a huge variety of conditions. While there are reviews that have summarized previous clinical trials pertaining to the use of cell-free therapy, we intend to highlight several clinical studies that are recently published at the time of this writing (Table 3). Unfortunately, to date, the results from many of these clinical trials have yet to be published.

**Figure 4:**
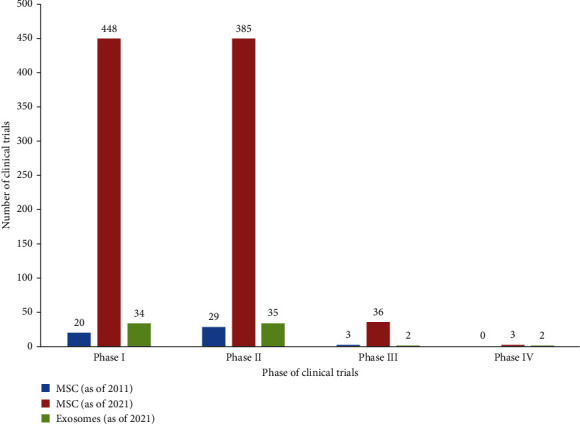
Number of mesenchymal stem cell (MSC) and exosome clinical trials between 2011 and 2021 by clinical phase (source: clinicaltrials.gov [accessed 5/5/2021]).

Overall, stem cell therapy has a longer history compared to cell-free therapy. A review on the stem cell clinical trials was published in the year 2011 [156]. One decade has passed since then, and a significant change in the current trend of stem cell clinical trials has been observed, most noticeably, the quantity (Figure 4). In 2011, 123 clinical trials using MSCs were recorded. Although some of the studies are in the combination of phase I/II, the majority are in phase II. The quantity of MSC clinical trials has grown tremendously, circa 25 times since the past decade. Notwithstanding, a total of 152 clinical trials using exosomes have also been recorded in the last 10 years. Although there is a huge surge in the number of clinical trials on MSCs and exosomes, the disease treated has not varied significantly and most of which are chronic diseases and disorders. While it is too hasty to draw a conclusion of the efficacy of cell-based therapy, the early observations of these trial results demonstrated that it is safe and feasible.

However, the clinical applications of MSCs or secretome are not without risks. Several pertaining concerns are promoting the growth of cancerous cells and nonspecific and undesirable differentiation of the transplanted cells at the target tissue. Perhaps, the most relevant risk of stem cell therapy is the malignant transformation of the administered cells. Many researchers have reported genomic instability in MSCs at higher passage [157, 158]. Thus, genotyping might be relevant to ensure the safety of the cells before transplantation.

## 7. Stem Cell vs. Secretome

### 7.1. Manufacturing

As a cellular product, the cell source poses the first major challenge to reproducibly manufacture clinically effective stem cells and secretome products. Stem cell manufacturing has been critically reviewed and discussed in the previous publication [159]. The production of stem cells is indeed a quite straightforward process. The stem cells can be grown on a large scale using bioreactors or large cell culture flasks under specific culture conditions [160]. Large-scale expansion is crucial to produce enough cells for downstream clinical application. Human platelet lysate (HPL) is often recommended as an alternative to fetal bovine serum (FBS) for good manufacturing practize- (GMP-) compliant stem cell expansion. Generally, stem cells cultured with HPL are smaller in size, display a tighter spindle-shaped morphology, and increased cell growth [161]. In addition, a chemically defined serum-free medium also can be used to replace the serum-based medium to avoid the batch-to-batch variation bothering the serum-based medium.

The manufacturing of clinically effective secretome is not an easy process. Notably, the quality and quantity of secretome are greatly influenced by the cell source and culture condition. Although secretome has been proven to work as effective as stem cells, nevertheless, it does not guarantee that the secretome harvested could work in the same way or as effective as the cultured cells. In vivo, transplanted stem cells produce secretome that could regenerate/repair the tissue or modulate the immune function in response to the signalling from the surrounding tissue. In contrast, this does not happen when the cells are grown in the laboratory. Therefore, it might be necessary to customize the culture condition that mimics the pathophysiological environment to produce clinically effective secretome [162].

When an appropriate cell source of clinically effective secretome is identified, the consistency of the cell source for all subsequent batches of secretome production must be addressed. This could be achieved by pooling the cells from the same tissue source of different healthy donors to produce multiple batches of secretome, mitigating the challenges of biological variation in tissue sources and donors [163]. Due to the finite replication of the primary cells, a more practical approach is to use “immortalized” cell lines or PSC-derived stem cells. Although studies have reported that the regenerative properties of secretome [164] and sEVs [165] harvested from immortalized stem cells are not compromised. Nevertheless, all these must be carefully investigated to ensure that the immortalized cells are stable and continue to produce secretome products that are bioequivalent to those from nonimmortalized parental cells. In addition, secretome enrichment protocols could also be employed to enhance the production of secretome in the laboratory [166]. It should be taken into consideration that the type of media used to harvest secretome may also affect the quality and efficacy of secretome. To avoid interference from HPL, basal media is often employed to harvest the secretome to determine the efficacy of stem cell secretome. Nevertheless, the sudden switch from nutrient-rich to basal media may change the cell's behavior and subsequently modifying the secretome profile. Would the basal media harvested secretome work as effective as the stem cells in the host and whether the secretome harvested from cells cultured in complete growth media might have better clinical efficacy compared to the secretome harvested from basal media are some of the questions that remain to be answered. Based on the abovementioned arguments, the quality of secretome preparation is dependent on the source, culture condition, and secretome enrichment protocol. Therefore, secretome manufactured using different protocols may have different disease-relevant potency.

### 7.2. Quality Control

Quality control is crucial to ensure the safety and efficacy of cell-based products. Adherence to the GMP regulations assures the identity, strength, quality, and purity of the products. Strict adherence to the quality management system helps to prevent contamination, mix-up, deviation, failure, and error during production [167]. It is important to note that a lot of efforts have been given to establish GMP facilities to produce cellular products for clinical applications [168]. Apart from the GMP facility, guidelines are in place to characterize the stem cells. For instance, MSCs should be characterized according to the guideline recommended by the International Society of Cellular Therapy (ISCT) [169].

The quality control for secretome is way more complex compared to the stem cells. As the secretome is a mixture of EVs and soluble proteins, it is challenging to identify the active components from this mixture and hence, more efforts are required to characterize the secretome. For instance, proteomic analysis is needed to identify the type of proteins and their concentration in the preparation [96, 170]. For EVs, particularly small EVs (sEVs), it needs to be characterized according to the guidelines published by the International Society of Extracellular Vesicles (ISEV). The identification of size and number would require either zetasizer or nanoparticle tracking analysis (NTA). Notably, quantifiable metrics defining the identity of sEV preparations should reflect the cellular origin of the sEVs in preparation, the presence of lipid membrane vesicles, and the degree of physical and biochemical integrity of the vesicles. The combination of these metrics could quantify the identity of sEVs and facilitate stratification and comparison of different secretome preparations [77, 171]. As EVs contain miRNAs, the molecular technique is also required to characterize the miRNA profile [172, 173].

### 7.3. Cost of Production and Treatment

There is an argument whether the cost of production and treatment is lower in secretome therapy in comparison to stem cell therapy. For stem cell therapy, the number of stem cells that could be isolated from patients/donors is low; therefore, the harvested cells are usually expanded in the laboratory to attain enough cells for clinical applications. The process can take weeks to months. During the cell expansion, media change is typically done every 3-4 days. The high volume of spent media means to be discarded is a potential source of secretome that can be used clinically after proper processing. The preparation of cell-free therapies from the spent media can greatly reduce the cost of production. However, need to bear in mind that the secretome or exosomes collected form the spent media could have different biological components when the cells are cultured in different conditions. Thus, it is imperative to determine safety and efficacy as well as to characterize the secretome or exosomes secreted by cells cultured in different conditions. In another words, not all spent media can be processed to produce safe and effective secretome and exosomes.

Furthermore, as the secretome cannot self-replicate and have a short half-life in vivo, thus, the secretome might need to be given more frequently to exert its therapeutic effect. In contrast, stem cells can self-renew and survive in the body for a longer period. Stem cells can respond to the signaling molecules released by the injured cells by secreting the appropriate paracrine factors to stimulate tissue regeneration. On the other hand, the contents of secretome are already defined in vitro. Thus, preconditioning of the cells at the culture condition that mimic the disease pathophysiological condition might be needed to produce clinically relevant secretome. The used of specific instruments or biochemicals to replicate the disease pathophysiological condition in vitro will incur extra costs. Finally, the cost of secretome production is still likely to be higher than the cells as it requires extra concentration and purification steps [174].

### 7.4. Advantages and Disadvantages

The use of stem cells as regenerative medicine for various diseases has been progressing well since the past decade. However, the type of stem cell suitable for different diseases is still under vigorous debate since each stem cell subtype possesses its advantages and limitations. For instance, ESCs can differentiate into various types of tissue but its limitations, i.e., ethical dilemma, genetic instability, and teratocarcinoma, might overweight the benefits [175]. MSCs show several superior properties for therapeutic use compared to other types of stem cells, including easy to harvest and expand, both autologous and allogeneic cells can be used with minimal risk of rejection, free from ethical issues and have limited replicative lifespan, and hence have lower risks of malignant formation. However, MSCs are only capable to differentiate into certain lineages thus limiting their usage to only certain diseases [176]. Notably, stem cells could be differentiated into specific tissue as cell replacement therapy [177, 178]. This is the main advantage of stem cells over secretome. Another prominent property of stem cells is their ability to migrate to the site of injury (homing effect). Surprisingly, sEVs also possessed the homing ability. Studies showed that MSC-EVs were mainly accumulated in the inflamed kidneys [179] and injured brains [180, 181] in the acute kidney injury model and intracerebral haemorrhage models, respectively. The accumulation of systemically injected sEVs in the intracerebral hemorrhage model also showed that sEVs can cross the blood-brain barrier (BBB). Research also has indicated that stem cells could cross BBB. MSCs integrated into the endothelium through the adhesion molecules VCAM-1/VLA-4 and *β*1 integrin. After crossing the endothelial barrier, MSCs invade the host tissue via plasmic podia [182]. MSCs were also found to cross BBB through paracellular pathways that are normally inhibited by the presence of tight junctions [183]. These showed that both stem cells and secretome could be intravenously injected and reach the brain. Nevertheless, the bioavailability of these two subjects in the brain remains to be elucidated.

**Figure 5:**
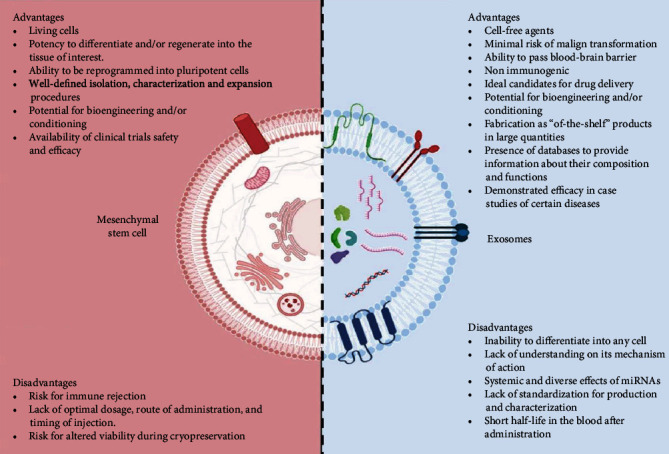
Advantages and disadvantages of MSCs and MSC-derived exosomes (created with BioRender.com).

Accumulating evidence suggests that secretome possesses many advantages over stem cell transplantation. Cell degeneration or senescence in the host after transplantation is not a concern for secretome therapy. It was also reported that secretome has lower cell surface proteins compared to stem cells, which makes allogeneic secretome safer than allogeneic stem cells because of the lower risk of immunogenicity [184]. Irreplicable property and absence of DNAs in secretome greatly reduce the risk of DNA mutation and tumor formation in the host. The use of secretome also reduces the possibility of vascular obstruction compared to larger stem cells. The bioactive components of secretome can be easily modulated by culturing the cells in different conditions. Secretome is also easier to store compared to stem cells, i.e., stem cells need to be stored in liquid nitrogen to maintain their viability while secretome can be stored in -20°C. Finally, the requirement to evaluate the safety and dosage of secretome is less stringent in comparison to the stem cells, making the journey to the clinical setting smoother and faster. This is because stem cells are living cells, and the fate of the transplanted cells is more difficult to predict.  Table 4 and Figure 5 summarize the comparison between stem cells and secretome from the perspective of manufacturing, quality control, cost of production, and treatment, as well as their advantages and disadvantages in clinical applications.

## 8. Future Perspective

Currently, cell-based therapies face two great challenges; how to anticipate decreased cell viability and biological functions during in vitro culture and how to prolong survival of transplanted cells. Consequently, several strategies can be envisaged to increase survival, immunomodulatory potential, and regenerative functions of cell-based therapy. Preconditioning, genetic modification, and tissue engineering are the dominant strategies. Furthermore, combinatorial approaches using nanotechnology could also improve the therapeutic performance of stem cells and secretome.

### 8.1. Stem Cells

#### 8.1.1. Genetic Modification

The combination of stem cell biology and genetic engineering has great potential in regenerative medicine. Through genetic modification, the researcher could induce or determine the cell's specific differentiation pathway after injection or enhance the adhesion potential of the stem cell to specific target. After transplantation, the fate of MSCs would be stochastically determined based on the microenvironment and biochemical stimulation of the host body; therefore, not all transplanted cells would contribute to the regeneration of damaged organs. As recently demonstrated in mice, transplanted MSCs could differentiate into osteoblasts in the heart [185]. Although cell differentiation can be achieved using the biochemical or biophysical stimulus in vitro, however, reverse differentiation may occur after transplantation or withdrawal of stimulants [186]. Therefore, genetic modification of the transplanted stem cells would be the key to achieve a directed and irreversible differentiation into the desired lineage. Several studies have been conducted on the therapeutic applicability of genetically modified MSCs in animal models of diabetes, myeloma bone disease, GvHD, and myocardial infarction.  Table 5 summarizes the modifiers, cell source, genetic engineering method, and applications from various studies.

In addition to the ability to differentiate, MSCs can be genetically engineered to home to the target tissue. For example, MSCs transduced with CXCR4 demonstrated higher homing in the mice model of myocardial infarction after intravenous administration [110, 235]. The overexpression of CXCR4 facilitated MSC aggregation and etching of collagenous tissue of the infarcted area [236]. Such strategies will help in the development of noninvasive cell therapies, since the route of administration is also important to avoid the formation of heterotopic tissue, especially in the case of genetically modified MSCs. On the other hand, poor cell survival after transplantation is a yet to resolved hurdle in MSC-based therapies. Studies show that genetic modification of MSCs with hypoxia-regulated heme oxygenase-1 (HO-1), Akt1, and Bcl-2 increased cell survival after transplantation in animal models by inhibiting cell apoptosis [236–238]. Thus, these strategies might be the possible solutions to increase the survival of MSCs after transplantation.

#### 8.1.2. Tissue Engineering Using MSCs

Another area of regenerative medicine is to combine cells and scaffolds to create a 3D implant. Tissue engineering seeks to recreate the in vivo environment to promote the development of tissues needed for transplantation. Various approaches have been studied, including protein-impregnated scaffolds [239], gene vector-incorporated templates [240], and cell-scaffold combinations (Table 6). Scaffolding alone has been shown to help repair certain types of damage [239]. However, incorporating MSCs into the scaffold improves the in situ repair process by acting as the precursors and stimulators [241].

Over the decades, much effort has been devoted to study the physical and chemical properties of various biomaterials, as these properties affect the differentiation pathway and adhesion capacity of MSCs. For example, the elasticity of a polyacrylamide matrix seeded with MSCs determines their differentiation pathway into neuronal, muscle, or bone lineage based on crosslink density [247]. Furthermore, studies indicated that the presence of carboxyl or hydroxyl groups on the scaffold surface prioritizes chondrogenic differentiation, while amino and sulfhydryl groups promote bone formation [248]. In addition to biometric properties, graft angiogenesis is another important factor in ensuring cell survival and therapeutic efficiency. The host's blood vessels can invade the transplant, but the process is very slow, and it takes weeks to vascularize just a few millimeters. Therefore, researchers incorporate angiogenesis-promoting factors such as endothelial progenitor cells (EPCs) and VEGF to hasten graft angiogenesis [249]. Unfortunately, no perfusion was observed upon implantation [250]. Currently, there are no established angiogenesis strategies available to support transplantation of large tissue due to delayed angiogenesis which resulted in cell apoptosis and necrosis. The approaches mentioned above only could increase the likelihood of angiogenesis.

### 8.2. Exosomes

#### 8.2.1. Preconditioning

**Figure 6:**
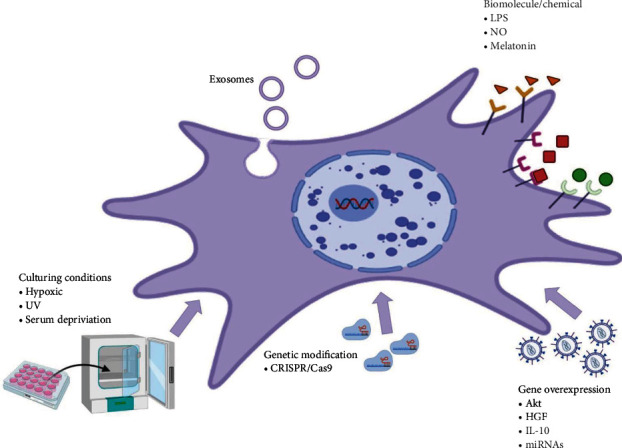
Preconditioning approaches to enhance the secretion and therapeutic efficacy of exosomes. The yield of secretome can be increased by preconditioning strategies such as introducing hypoxic and serum deprivation culture conditions and genetic modification using CRISPR technology as well as overexpression of certain genes. LPS: lipopolysaccharides; NO: nitric oxide; IL: interleukin; miRNAs: micro RNAs; UV: ultraviolet (created with BioRender.com).

Both 3D culturing and pretreatment of MSCs with cytokines, hypoxia, or chemicals are reliable methods to increase exosome secretion (Figure 6) [251]. In addition, MSC gene and cell surface modifications may be used to improve the therapeutic effect of exosomes.

*(1) Increasing Exosome Production*. Increasing the secretion of exosomes is an important but unmet process. Studies have shown that 3D culturing methods such as bioreactors and microcarriers could significantly increase the production of exosomes by cultured MSCs [252]. Generally, MSCs are processed on 2D surfaces in plastic dishes that do not reflect the physiological niches of MSCs. Therefore, the use of a 3D porous scaffold structure, such as beads, microfiber, or any other type of carrier is an attractive method to increase exosome production. One study showed that antifungal agents, i.e., imidazole and nitrefazole, significantly increase the production of exosomes in prostate cancer cells [253]. In this case, nitreprazole increased the level of the protein Rab27a, which regulates MVB exocytosis. Other chemicals, such as azole and pentetrazole, have also been shown to activate exosome biogenesis-related molecules, i.e., Alix and NSmas2. The techniques may be employed to increase the production of exosomes from MSCs by modulating the biogenesis and release of exosomes [253]. On the other hand, gene editing is another effective way to increase the production of exosomes. There are several important genes, such as phospholipase D2, that are important for the biosynthesis and secretion of exosomes, and the overexpression or dysfunction of these genes promotes exosome secretion. For example, the overexpression of phospholipase D2 led to a twofold increase in the number of secreted exosomes [254].

*(2) Hypoxia Preconditioning*. Hypoxia culture is commonly used to prime MSCs. Several studies found that exosomes derived from MSCs cultured in hypoxic condition showed greater angiogenic potential compared to exosomes secreted by MSCs cultured in normoxic condition [255]. The exosomes secreted by hypoxia primed MSCs were uptaken more effectively by the target cells compared to exosomes derived from MSCs cultured in normoxic condition. The uptaken exosomes promoted the VEGF expression and protein kinase A signaling pathway activation in the target cells, which resulted in improved angiogenesis [256, 257]. However, the reason for these phenomena is still unclear, and how different culture conditions influence the uptake of exosomes needs to be further investigated.

*(3) Chemical Preconditioning*. In contrast to hypoxic priming, the effects and mechanisms of biomolecule priming in exosomes are better studied. Various studies have compared the therapeutic effect between lipopolysaccharides (LPS) preconditioned and unconditioned exosomes. LPS conditioned exosome showed higher regeneration potential for liver disease preclinically by reducing the expression of IL-6 and TNF-*β* [258] and upregulated the expression of THP-1, which in turn stimulate the synthesis of more anti-inflammatory cytokines and contributed to the polarization of M2 macrophages [259]. A recent study has also shown that macrophages cultivated with exosomes from LPS-primed MSCs expressed higher levels of STAT3 gene, secretion of cytokines (IL-10 and IL-15), and growth factors (FLT-3 L) which play vital roles in cell regeneration and antiapoptosis [260]. Several other molecules have been tested as preconditioners, including thrombin to improve fibroblast proliferation, enhance anti-inflammatory effects, accelerate wound healing [176] and melatonin to increase BCL2, HO1, IL-10, and VEGF expression, and suppress the expression of various apoptosis-related genes such as ICAM1, HIF1, NFkB, and IL-1*β* in a rat model [261]. Exosomes derived from deferoxamine-primed MSCs contained higher levels of miR-126a that support angiogenesis [262].

#### 8.2.2. Genetic Modification

In 2010, a study reported that the paracrine factor secreted by MSC-overexpressed GATA-4 increased blood vessel formation and cell survival [263]. Next, in a mouse model of myocardial infarction, exosomes secreted by the genetically modified stem cells with GATA-4 were more effective in increasing angiogenesis and reducing the number of apoptotic cardiac cells compared to the exosomes secreted by native stem cells [264]. MSC-derived exosomes that overexpress GATA-4 and CXCR4 have been shown to contain cardioprotective antiapoptotic miR-19a that activates Akt and ERK signaling pathways [265, 266]. Similarly, exosomes from MSCs that overexpress SDF-1 have been shown to prevent apoptosis of cardiomyocytes and exhibit improved cardiac regeneration properties [267]. Genetic modification methods have also been investigated to improve the therapeutic potential of exosomes for musculoskeletal disorders, liver and lung disorders, and inflammation-related disorders.

#### 8.2.3. Combining Nanoparticles, Exosomes, and Stem Cells

Nanotechnology is the term used to cover the design, construction, and utilization of functional structures with at least one characteristic dimension measured in nanometers. In recent years, the application of nanotechnology in stem cells has made great advances. Currently, nanotechnology is utilized to control the proliferation and differentiation of the transplanted stem cells.

Carbon nanotubes (CNTs) are widely used in various fields, including medicinal chemistry, molecular electronics, and tissue engineering, due to their unique mechanical, physical, and chemical properties. CNTs can be designed and filled with DNA or peptide molecules to improve their properties and functions, such as biocompatibility and recognition capability in the molecular treatment of diseases [268–270]. In a study that examined the effect of CNTs on the proliferation and differentiation of human stem cells, the result showed that CNTs inhibit the proliferation of cells of the embryonic kidney cell line HK293 and reduce the adhesion efficiency of cells in a dose- and time-dependent manner, but similar CNTs can stimulate the formation of bumps on the surface of human osteoblasts and fibroblasts, which are one of the active cells in the immune response [271]. Nanomaterials such as CNTs have enormous potential in the field of regenerative medicine in several areas, including (1) the development of nanovehicles to deliver biomolecule-based products to MSCs and (2) the creation of new biomedical applications for electroactive CNTs in combination with MSCs. However, despite the immense potential of nanoparticles, the method of delivering nanoparticles to the target cells was still a major problem. The maximum size of particles entering cells is 25 nm to 700 nm; so, it is difficult for nanosized particles to penetrate cells due to the tension and adhesion strength of the cell surface. As an alternative, the nanoparticles can be bonded to the external cell membrane.

## 9. Conclusion

Regenerative medicine holds an immense potential for a variety of diseases in which there is a high unmet clinical need. Regenerative medicine covered a wide range of subbranches including cell and gene therapies and tissue engineering applications. Stem cells have been the focus for years because of their biological potential, and paracrine effect is the pivotal mechanism in stem cell-based tissue regeneration. Thus, cell secretome has attracted great attention as therapeutics in recent years and has been suggested as alternative to stem cell therapy as cell-free agents. The high degree of confidence in cell-based therapy is vividly indicated by the significant increase in the number of ongoing and planned clinical trials worldwide. Despite the relatively slow rate of translational success from laboratory to clinics, expectations, optimism, and excitement surrounding this field remain great.

## Figures and Tables

**Table 1:** Recent studies applied secretome and its components via intravenous (IV) route.

Cell type	Disease model	Application method	References
Cardiac stem cells	Cardiac myopathy	IV injection of 30×10^9^ exosomes	[122]
BMSCs	Pulmonary hypertension	IV injection of culture media (30 *μ*g/100 *μ*L)	[123]
BMSCs	Asthma	IV injection of culture media (500 *μ*g/mL)	[124]
BMSCs	Lung fibrosis	IV injection of 10 *μ*g EVs	[125]
MSCs	Myocardial inflammation	IV injection of 200 *μ*L of 300 *μ*g exosomes	[126]
iPSCs	Limb ischemia	IV injection of 200 *μ*g exosomes	[127]
hMSC544 cells	Ovarian cancer	IV injection of 100 *μ*L exosomes	[128]
Schwann cells	Peripheral neuropathy caused by type 2 diabetic	IV injection of 200 *μ*L exosomes	[129]
BMSCs	COVID-19	IV injection of 15 mL exosomes	[130]

**Table 2:** Completed clinical trials that evaluated the safety, efficacy, and feasibility of stem cell therapy.

Type of stem cell	Treated disease	Trial design	Number of cells	Route of administration	Outcomes	References
Pluripotent stem cells	Ischemic heart disease	An open label, phase I study to assess both the feasibility and safety of epicardial delivery of a fibrin gel embedded with human embryonic stem cell- (ESC-) derived CD15+ IsI-1+ progenitors	NA	Epicardial transplantation of cells embedded in fibrin patch	Not yet available	NCT02057900
	Advanced Stargardt's macular dystrophy	A follow-up of a phase I/II, open-label, nonrandomized, 4-cohort, dose escalation, multicenter clinical trial to evaluate the long-term safety and tolerability of human ESC-derived retinal pigment epithelium (hESC-RPE) cellular therapy	0.05 × 10^6^ to 0.2 × 10^6^ hESC-RPE/kg	Subretinal injection	Not yet available	NCT02941991
	Stargardt's macular dystrophy	A phase I/II, open-label, nonrandomized, sequential, multicenter clinical trial to evaluate the safety and tolerability of hESC-RPE cellular therapy in patients with SMD and to evaluate potential efficacy endpoints to be used in future studies of hESC-RPE cellular therapy	0.05 × 10^6^ to 0.2 × 10^6^ hESC-RPE/kg	Subretinal injection	Not yet available	NCT01469832
	Outer retinal degenerations	A phase I/II, open label, nonrandomized, prospective study to determine the safety of hESC-RPE subretinal injection	0.1 × 10^6^ hESC- RPE/kg	Subretinal injection	Not yet available	NCT02903576
	Stargardt's macular dystrophy	A phase I/II, open-label, nonrandomized clinical trial to evaluate the safety and tolerability of subretinal injection of hESC-RPE	0.05 × 10^6^ to 0.2 × 10^6^ hESC-RPE/kg	Subretinal injection	Not yet available	NCT01345006
	Age-related macular degeneration	A phase I/II, open-label, nonrandomized, sequential, multicenter clinical trial to evaluate the safety and tolerability of subretinal injection of hESC-RPE	0.05 × 10^6^ to 0.2 × 10^6^ hESC-RPE/kg	Subretinal injection	Not yet available	NCT01344993
	Age-related macular degeneration	A phase I/II trial, open-label, nonrandomized study to evaluate the long-term safety and tolerability of MA09-hRPE cellular therapy in patients with advanced dry age-related macular degeneration from one to five years following the surgical procedure to implant the MA09-hRPE cells	0.05 × 10^6^ to 0.2 × 10^6^ hESC-RPE/kg	Subretinal injection	Not yet available	NCT02463344
	Advanced Stargardt's macular dystrophy	A phase I/II trial, open-label, nonrandomized study to evaluate the long-term MA09-hRPE cellular therapy in patients with advanced Stargardt's macular dystrophy from one to five years following the surgical procedure to implant the MA09-hRPE cells	0.05 × 10^6^ to 0.2 × 10^6^ hESC-RPE/kg	Subretinal injection	Not yet available	NCT02445612
Multipotent stem cells	Diabetic peripheral neuropathy	A phase II study to investigate the effects of mesenchymal stem cell (MSC) transfusion	NA	Intravenous infusion	Not yet available	NCT02387749
	Idiopathic pulmonary fibrosis	A phase 1b study to evaluate the safety and feasibility, particularly with respect to adverse acute hemodynamic effects and profibrosis of MSC treatment	1 × 10^6^ or 2 × 10^6^ hMSCs/kg	Intravenous infusion	Feasible and satisfactory short-term safety profile.	[141] NCT01385644
	Acute respiratory distress syndrome	A phase 1 clinical trial to test the safety of a single dose of allogeneic bone marrow-derived MSCs (BMSCs)	1 × 10^6^, 5 × 10^6^, or 10 × 10^6^ of hMSCs/kg	Intravenous infusion	Safe and well tolerated	[142] NCT01775774
	Periapical periodontitis	A randomized, controlled phase I/II clinical trial to evaluate the safety and efficacy of human umbilical cord-derived mesenchymal stem cells (UC-MSCs) encapsulated in a plasma-derived biomaterial for regenerative endodontic procedures in mature permanent teeth with apical lesions	1 × 10^6^ cells/biomaterial	Encapsulated in platelet poor plasma	Safe and effective	[143] NCT03102879
	Cleft lip and palate	A phase I trial to evaluate the feasibility and safety of deciduous dental pulp stem cells	1 × 10^6^ cells/biomaterial	Embedded in hydroxyapatite-collagen sponge	Feasible and safe	[144] NCT01932164
	Knee osteoarthritis	A multicentre, phase I/II clinical trial to evaluate the clinical use of allogenic BMSCs	40 × 10^6^ cells/kg	Intra-articular injection	Feasible and safe	[145] NCT01586312
	Knee osteoarthritis	A phase 2 study to determine the clinical response to autologous bone marrow aspirate concentrate and platelet-rich plasma injection	NA	Intra-articular injection	Not yet published	NCT02958267
	Frailty	A phase I/II randomized, double-blind, placebo-controlled clinical trial to evaluate the safety of MSC therapy	100 × 10^6^ cells/kg	Intravenous infusion	Safe	[146] NCT02065245
	Retinal degeneration	A phase 1 study to report electroretinographic (ERG) findings in advanced glaucoma treated with a single intravitreal injection of BMSCs	1 × 10^6^ cells/kg	Intravitreal injection	Not yet available	NCT02330978
	Spinal cord injury	A phase I/II study to evaluate the safety and efficacy of intrathecal administration of allogeneic UC-MSCs to patients with spinal cord injury	1 × 10^6^ cells/kg	Intrathecal injection	Not yet available	NCT02481440
	Knee osteoarthritis	A phase I/II study to evaluate the feasibility and safety of the implantation of 40 million BMSCs in knees with osteoarthritis of grade II-IV (Kellgren and Lawrence)	40 × 10^6^ cells/kg	Intra-articular injection	Not yet available	NCT01183728
	Chronic ischemic cardiomyopathy	A phase II trial to assess the feasibility, safety, and efficacy of *trans*-endocardial administration of autologous MSCs and cardiac progenitor cells (CPCs), alone, and in combination	150 × 10^6^ cells/kg b.w.	*Trans*-endocardial injection	Feasible, safe, and effective	[147] NCT02501811
	Cardiomyopathy	A phase I study to examine the safety and feasibility of allogeneic human MSCs by *trans*-endocardial injection to cancer survivors	100 × 10^6^ cells/kg	*Trans*-endocardial injection	Safe and feasible	[148] NCT02509156
	Ischemic cardiomyopathy	A phase 1/2 randomized comparison study to test whether allogeneic MSCs are as safe and effective as autologous MSCs	20 × 10^6^, 100 × 10^6^, or 200 × 10^6^ cells/kg	*Trans*-endocardial injection	Safe and effective	[149] NCT01087996
	Nonischemic dilated cardiomyopathy	A phase I/II study to assess the safety of autologous and allogeneic human MSC administration	100 × 10^6^ cells/kg	Trans-endocardial injection	Safe	[150] NCT01392625
	Myocardial infarction	A phase II trial to compare the safety and efficacy of 2 doses of allogeneic BMSCs	20 × 10^6^ or 100 × 10^6^ cells/kg	*Trans*-endocardial injection	Safe	[151] NCT02013674
	Ischemic cardiomyopathy	A phase I/II study to demonstrate the safety of *trans*-endocardial injection of autologous MSCs and bone marrow mononuclear cells	100 × 10^6^ or 200 × 10^6^ cells/kg	*Trans*-endocardial injection	Safe	[152] NCT00768066
	End-stage liver disease	A phase I/II study to investigate the feasibility, safety, and efficacy of autologous MSC injection	3 − 5 × 10^6^ MSCs	Peripheral or portal vein injection	Feasible, safe, and effective	[153] NCT01440309
	Knee osteoarthritis	A randomized, phase 2b study to assess the efficacy and safety of a single intra-articular injection of adipose tissue-derived MSCs (AT-MSCs)	1 × 10^8^ MSCs	Intra-articular injection	Safe and effective with satisfactory functional improvement and pain relief in patients	[154]
	Heart failure	A phase 1/2 randomized controlled trial to evaluate the safety and efficacy of the intravenous infusion of UC-MSCs in patients with chronic stable heart failure and reduced ejection fraction	1 × 10^6^ MSCs/kg	NA	Safe and effective with improvement in quality of life	[155] NCT01739777
	COVID-19	A phase 1 clinical trial to evaluate the safety of human UC-MSC infusion in the treatment of patients with moderate and severe COVID-19 pulmonary disease	3 × 10^7^ of UC-MSCs	NA	Safe and well tolerated without serious adverse events	[140]

NA: not available.

**Table 3:** Cell-free treatment in clinical trials for various diseases.

Target disease	Cell	Clinical trials identifier	Administration	Dosage	Results
COVID-19	MSC-derived exosomes	NCT04491240	Inhalation	0.5 − 2 × 10^10^ exosomes	No observable side effects in 30 days. Improvement in overall treatment is not insignificant compared to standard therapy.
Chronic ulcer	MSC conditioned media	NCT04134676	Topical	Unknown	Not available
Keloid	Umbilical cord-MSC conditioned medium	NCT04326959	Intralesional	1 mL/cm^3^	Not available
Knee osteoarthritis	MSC conditioned medium	NCT04314661	Intra-articular injection	2 mL 2 weeks after 5 × 10^5^ MSC cells	Not available
SARS-CoV-2-associated pneumonia	MSC-derived exosomes	NCT04276987	Inhalation	2.0 × 10^8^ vesicles/3 mL for 5 days	Not available
Multiple organ failures after surgical repair of aortic dissection	MSC-derived exosomes	NCT04356300	Intravenous	150 mg exosomes for 2 weeks	Ongoing
Chronic low back pain	Platelet-rich plasma with exosomes	NCT04849429	Intrathecal	2 mL	Ongoing
Cerebrovascular disorders	MSC-derived exosomes	NCT03384433	Intravenous	Not available	Ongoing
COVID-19	MSC conditioned medium	NCT04753476	Intramuscular	0.5-1 mL (3 doses)	Ongoing

**Table 4:** Comparison between stem cell and secretome.

Aspect	Stem cell	Secretome
Manufacturing	(i) General culture condition is normally used but special culture condition might be needed to produce specific cells (e.g., chondrogenic media to produce chondrogenic-differentiated MSCs) (ii) The consistency of the cell source has to be maintained for allogeneic stem cells (iii) May contain elements of external sources (FBS, HPL) (iv) Require a large number of cells for clinical applications	(i) General culture condition can be used but special culture condition mimicking the pathophysiological condition of the target diseases might be needed to produce the “bioequivalent” secretome (ii) The consistency of the cell source has to be maintained (iii) Enrichment protocol might be needed to enhance the production of secretome (i) Secretome may contain elements of external sources (FBS, HPL) (ii) High volume of media is collected, and it needs to be processed and concentrated for clinical applications
Quality control	(i) Stem cell markers are well established (ii) The characterization techniques are well established (iii) Specific functionality assay is needed to determine the efficacy	(i) The characterization is complex since secretome contain many elements such as growth factors, cytokines, and extracellular vesicles (ii) Specific functionality assay is needed to determine its efficacy
Cost of production and treatment	(i) Cost can be reduced via large-scale expansion of allogeneic stem cells (ii) Treatment dose is easier to be justified by number of cells	(i) Repetitive collection of secretome from spent culture media can greatly reduce the cost of production (ii) Extra cost is needed for downstream processing of secretome (concentration and purification) (iii) Treatment dose is vague (protein amount vs number of particles) (iv) It is unsure which component(s) of the secretome are exerting therapeutic effects
Advantages	(i) Stem cells can be differentiated into specific lineages to improve the therapeutic efficacy and treat different diseases (ii) Mesenchymal stem cells are easy to isolate and expand, have low immunogenicity (both autologous and allogeneic cells can be used clinically), free from ethical issues, and have limited replicative lifespan, hence safe from malignant formation (iii) Can be reprogrammed into pluripotent stem cells (PSCs) (iv) Can cross blood-brain barrier (BBB) (v) Can migrate and home to the target tissue in response to the signal release by the injured cells (vi) Living cells can exert the therapeutic effects for a longer period. Thus, less frequency of administration is needed (e.g., once in every 6 months)	(i) The therapeutic components of the secretome could be customized by modifying the culture condition (preconditioning) (ii) Can cross BBB (iii) Can circulate and home to the target tissue (iv) Low risk of mutation, carcinogenesis, and immunogenic as they are not living cells (v) Lower risk of vascular obstruction as they are smaller in size compared to stem cells (vi) Easier to store (vii) Cell degeneration or senescence in the host after transplantation is not a concern
Disadvantages	(i) Higher risk of mutation and carcinogenesis (especially the PSCs) (ii) Ethical issue (embryonic stem cells) (iii) Might illicit host immune response to reject the transplanted cells (especially the allogeneic stem cells) (iv) Cell degeneration or senescence in the host after transplantation (v) Potential vascular obstruction (vi) More stringent storage condition to maintain the cell viability (vii) More optimization is needed to improve the safety and efficacy (e.g., optimum dosage and route of administration)	(i) Cannot be used as cell-replacement therapy and relying on the proliferation of host cells for tissue regeneration (ii) Lack of understanding on its mechanism of action (iii) Lack of long-term safety data (iv) Lack of standardization (v) Short half-life in the body. Thus, might need more frequent administration (vi) Difficult to purify the specific therapeutic components (e.g., exosomes). Thus, the secretome products are highly heterogeneous

**Table 5:** Genetic modifications in human MSCs and the disease models tested.

Factor overexpressed	MSC source	Method	Disease	Reference
Akt	Human umbilical cord	Adenovirus	Acute myocardial infarction	[187]
Angiotensin II type 2 receptor	Human bone marrow	Lentivirus	LPS-induced acute lung injury	[188]
Arginine decarboxylase	Human adipose tissue	Retrovirus	Spinal cord injury	[189]
Basic fibroblast growth factor (bFGF)	Human bone marrow	Lentivirus	Angiogenesis	[190]
Brain-derived neurotrophic factor (BDNF)	Human umbilical cord blood	Plasmid transfection	Neurological injury and disease	[191]
	Human bone marrow	Lentivirus	Neuronal degeneration	[192]
C-C chemokine receptor type 2 (CCR2)	Human bone marrow	Lentivirus	Ischemic stroke	[193]
CXC chemokine receptor 4 (CXCR4)	Human umbilical cord	Lentivirus	Radiation- induced lung injury	[194]
Cytosine deaminase (CD) and herpes simplex virus thymidine kinase (HSV-tk)	Human umbilical cord blood	Lentivirus	Ovarian cancer	[195]
Ephrin-B2	Human bone marrow	Plasmid transfection	Ischemic tissues	[196]
Forkhead box protein (Foxa2)	Human adipose tissue	Plasmid transfection	Acute liver injury	[197]
Glial-derived neurotrophic factor (GDNF)	Human adipose	Lentivirus	Renal interstitial fibrosis	[198]
	Human bone marrow	Adenovirus	Nephrotoxic serum nephritis	[199]
Glucocorticoid-induced tumour necrosis factor-related receptor (GITR)	Human bone marrow	Plasmid transfection	Small cell lung cancer	[200]
Granulocyte chemotactic protein-2 (GCP-2)	Human adipose tissue	Lentivirus	Myocardial infarction	[201]
Heme oxygenase-1 (HO-1)	Human embryonic stem cell	Lentivirus	Myocardial infarction	[202]
Hepatocyte growth factor (HGF)	Human bone marrow	Retrovirus	Bladder outlet obstruction	[203]
	Human umbilical cord	Lentivirus	Myocardial infarction	[204]
	Human bone marrow	Adenovirus	Liver fibrosis	[205]
	Human umbilical cord	Adenovirus	Injured sinonasal mucosa	[206]
	Human umbilical cord	Adenovirus	Parkinson's disease	[207]
	Human umbilical cord blood	Plasmid transfection	Liver fibrosis	[208]
	Human bone marrow	Lentivirus	Spinal cord injury	[209]
Hepatocyte nuclear factor 4a (HNF 4a)	Human umbilical cord	Lentivirus	Hepatocellular carcinogenesis	[210]
Human N-cadherin	Human umbilical cord blood	Lentivirus	Myocardial infarction	[211]
Hypoxia inducible factor-1a (HIF-1a)	Human bone marrow	Lentivirus	Angiogenesis	[212]
IL-4	Human adipose tissue	Lentivirus	Multiple sclerosis	[213]
IL-10	Human amniotic fluid	Human amniotic fluid	Liver fibrosis	[214]
	Human bone marrow	AAV	Acute ischemic stroke	[215]
Leptin	Human bone marrow	Lentivirus	Myocardial infarction	[216]
LIM-homeobox transcription factor islet-1 (ISL1)	Human bone marrow	Lentivirus	Myocardial infarction	[217]
miR-101-3p	Human bone marrow	Lentivirus	Oral cancer	[217]
miR-16-5p	Human bone marrow	Plasmid transfection	Colorectal cancer	[218]
miR-199a	Human bone marrow	Plasmid transfection	Glioma	[218]
miR-199a-3p	Human bone marrow	miRNA transfection	Renal ischemia/reperfusion injury	[219]
miR-let-7d or miR-154	Human bone marrow	Lentivirus	Lung injury	[220]
miRNA-181	Human umbilical cord blood	Lentivirus	Myocardial ischemia-reperfusion injury	[221]
Neuregulin 1 (NRG1)	Human adipose tissue	Adenovirus	Cerebral ischemia	[222]
Nuclear factor (erythroid-derived 2)-like 2 (Nrf2)	Human amniotic fluid	Lentivirus	Acute lung injury	[194]
Oct4 and Sox2	Human adipose tissue	Plasmid transfection	Liver injury	[223]
PARKIN	Human Wharton's jelly	Plasmid transfection	Parkinson's disease	[224]
Pigment epithelial-derived factor (PEDF)	Human bone marrow	Lentivirus	Hepatocellular carcinoma	[225]
SRC3-specific short hairpin RNA (sh-SRC3)	Human bone marrow	Lentivirus	Multiple myeloma	[226]
sST2	Human adipose tissue	Lentivirus	Occupational asthma	[227]
	Human adipose tissue	Lentivirus	Endotoxin-induced acute lung injury	[228]
Thioredoxin-1 (Trx-1)	Human umbilical cord	Adenovirus	Acute radiation injury	[229]
Tissue matrix metalloproteinase inhibitor 2 (TIMP2)	Human umbilical cord	Lentivirus	Myocardial infarction	[230]
TNF-related apoptosis-inducing ligand (TRAIL)	Human bone marrow	Lentivirus	Non-small-cell lung cancer	[231]
	Human adipose tissue	Plasmid transfection	Non-small-cell lung cancer	[232]
	Human adipose tissue	Lentivirus	Glioblastoma multiforme	[233]
Transforming growth factor b1 (TGF-b1)	Human bone marrow	Lentivirus	Angiogenesis	[190]
Vascular endothelial growth factor (VEGF)	Human bone marrow	Lentivirus	Peripheral nerve injury	[234]

**Table 6:** Tissue engineering therapies using MSCs.

Disease	Study organism	Cell	Scaffold	Outcome	Reference
Osteochondral defect	Rabbit	Autologous MSCs	Injectable synthetic ECM	Cartilage filled the full-thickness defect	[242]
Spinal cord injury	Rat	Autologous MSCs	Hydrogels	Enhanced ingrowth of axons in the lesion and improvement in function	[243]
Critical size bone defect	Mouse	OSX-modified murine MSCs	Collagen sponge	Enhanced bone formation	[244]
Tendon defect	Rat	C3H10T1/2 cells stably transfected with BMP-2 and active Smad8 variant	Collagen sponge	Tendon regeneration	[245]
Articular cartilage defect	Rabbit	Autologous MSCs modified with TGF-1	Chitosan scaffold	Enhanced repair; defect filled with hyaline cartilage	[246]

## Data Availability

No data were used to support this study.
